# Examination of the lung and lymphoid tissue mRNA transcriptome response in dairy calves following experimental challenge with bovine alphaherpesvirus one (BoHV-1)

**DOI:** 10.1371/journal.pone.0319575

**Published:** 2025-05-02

**Authors:** Stephanie O’Donoghue, Bernadette Earley, Dayle Johnston, Matthew S. Finnie, S. Louise Cosby, Ken Lemon, Michael J. McMenamy, Jeremy F. Taylor, Jae Woo Kim, Derek W. Morris, Sinéad M. Waters

**Affiliations:** 1 Animal and Bioscience Research Department, Animal and Grassland Research and Innovation Centre, Teagasc, Grange, Meath, Ireland; 2 School of Biological and Chemical Sciences, University of Galway, Galway, Ireland; 3 Veterinary Sciences Division, Agri-Food and Biosciences Institute, Stormont, Belfast, Ireland,; 4 Division of Animal Sciences, University of Missouri, Columbia, Missouri, United States of America; University of Messina: Universita degli Studi di Messina, ITALY

## Abstract

Bovine alphaherpesvirus one (BoHV-1) is a primary cause of bovine respiratory disease (BRD), and a leading cause of morbidity and mortality in cattle. The transcriptomic responses of key respiratory and immune associated tissues of dairy calves following experimental challenge with BoHV-1 are unknown. Thus, the study objective was to examine the gene expression profiles of multiple tissue types from dairy calves following an infectious challenge with BoHV-1. Holstein-Friesian bull calves (mean age ±  SD 149.2 days ±  23.8; mean weight ±  SD 174.6 kg ±  21.3 kg were challenged with either BoHV-1 inoculate (6.3 ×  10^7^/mL ×  1.35mL) (n =  12) or sterile phosphate buffered saline (n =  6). Animals were euthanised on day 6 post-challenge and tissue samples collected, including bronchial (BLN) and mediastinal lymph nodes (MLN), pharyngeal tonsil (PGT) and healthy (HL) and lesioned right cranial lung (LL). Total RNA was extracted and libraries sequenced on an Illumina NovaSeq 6000. Differential expression analysis was conducted using edgeR and pathways analysed using DAVID. A weighted gene co-expression network analysis (WGCNA) was conducted separately for each tissue type to identify networks significantly associated with BoHV-1 infection. Differentially expressed genes (DEGs) were identified in all tissues (P <  0.05, FDR <  0.1, FC >  2). Thirty-three DEGs were common to all tissues and enriched pathways included Influenza A and Herpes simplex 1 infection (P < 0.05, FDR < 0.05). Modules enriched for antiviral and innate immune processes were identified for each tissue type. Of the 33 DEGs common to all tissues, 26 were also identified as hub genes in the blood (blue) module. Our use of a controlled experimental challenge allowed for improved understanding of the immune response of dairy calves to a BoHV-1 infection. Furthermore, discovering DEGs that are common to all tissues, including whole blood, indicates future focus areas in research surrounding BRD diagnostic biomarkers.

## Introduction

Bovine respiratory disease (BRD) is a multifactorial syndrome affecting cattle of all ages in beef and dairy industries not only in Ireland [1,2] but internationally [3], often responsible for reduced animal performance and economic losses [4]. Stress resulting from management and environmental factors as well as infections caused by bacterial and viral agents influence the onset of BRD. A key BRD-associated virus, BoHV-1 is an *alphaherpesvirus* which causes the clinical disease infectious bovine rhinotracheitis (IBR) in cattle [5]. BoHV-1 has been shown to remain latent within the sensory neurons within trigeminal ganglia [6] and also in the pharyngeal tonsil of infected cattle [7]. As a result, despite recovering from acute infection, the dormant virus can reactivate causing the emergence of symptoms and viral shedding [8,9]. Although vaccines may reduce clinical symptoms of IBR, they have not been fully successful at preventing infection with BoHV-1 or the establishment of BoHV-1 latency [5].

Next-generation sequencing methodologies have been utilised in efforts to gain an improved understanding of the response of animals to infection by BRD pathogens at the molecular level. RNA sequencing (RNA-Seq) has been used to analyse the gene expression profiles of lung, pharyngeal tonsil, retropharyngeal lymph node, nasopharyngeal lymph node [10] and bronchial lymph node [11] in beef steers following experimental challenge with BoHV-1, resulting in the identification of key genes influencing the activation of key immune networks across lymphoid tissues. We have previously identified key biological pathways and processes such as inflammatory response, and viral pathways such as Influenza A and defence response to virus in the whole blood transcriptomes of calves experimentally challenged with BoHV-1 [12]. Furthermore, other studies have utilised a network-based approach to analyse the blood transcriptome profiles of cattle with BRD and have found networks and hub genes involved in the immune response to infection [13–15]. However, in these studies the BRD causal pathogen was unknown. One possible application for the discovery of important genes related to the immune response in whole blood is the creation of a BRD diagnostic that can be used “ante-mortem”.

The mechanisms by which BoHV-1 establishes infection and the host response to disease at the molecular level requires further study in weaned dairy calves. In particular, gene and network regulation in respiratory and immune tissues should be studied to complement findings in whole blood [12]. Studies focusing on pan-tissue transcriptomics in response to key pathogens allow for a better understanding of the immune system functionality at the molecular level. Furthermore, the use of network based approaches allows for the examination of interactions between genes governing a particular phenotypic outcome [16], and the identification of hub genes involved in the regulation of other network genes, may enable the development of biomarkers for traits such as BRD [17]. Hub genes are highly connected genes within a co-expression module that help to explain the module functioning [14,18].

The objectives of this study were threefold; first, to characterise the gene expression profiles of key immune and respiratory associated tissues in dairy calves experimentally infected with BoHV-1 and identify the biological processes that are enriched. Second, to cross-analyse the DEGs identified with those previously identified in whole blood from the same animals in response to a BoHV-1 challenge [12]. Finally, to perform WGCNA on the analysed tissues augmented by the whole blood data from O’Donoghue *et al*. (2023) and to determine if any modules and associated pathways identified in the key respiratory and immune tissues are also detected in the blood.

## Materials and methods

### Animal model

All animal studies were carried out in accordance with the UK Animals (Scientific Procedures) Act 1986 and with the approval of the Agri-Food and Biosciences Institute Northern Ireland Ethical Review Committee. The details of the study have been reported in accordance with the ARRIVE guidelines (https://arriveguidelines.org/).

The model and sampling protocol used in this study have previously been described (O’Donoghue *et al*., 2023). In brief, Holstein-Friesian bull calves (mean ±  SD age 149.2 ± 23.82 days) were selected based on low BoHV-1 specific maternally derived antibody levels and negative BoHV-1 PCR status. Calves were either challenged with BoHV-1 inoculum (strain 2011-426) (dose =  6.3 ×  10^7^/mL in a volume of 1.35mL per animal) (n =  12) or mock-challenged with sterile phosphate buffered saline (PBS) (n =  6). Clinical signs such as nasal discharge, ocular discharge, general demeanour, size of mandibular lymph nodes, presence of a cough, respiratory rate, respiratory character, mouth breathing, dyspnoea, presence of an expiratory grunt and rectal temperature were recorded daily from the day of challenge until euthanasia, and scores were calculated by a trained veterinarian blinded to the calves treatment status, using the scoring system described by Johnston *et al*. (2019). On day 6 post-challenge, calves were euthanised by captive bolt. At post-mortem, a trained veterinary pathologist examined and scored the lungs using an AFBI standardised scoring system, which assigns the percentages of lesions present on the total lung area and on component parts of the lungs. The workspace and implements were thoroughly cleaned and disinfected with bleach and 75% ethanol and sprayed with RNaseZap for RNase inhibition, before tissue collection and between the processing of each animal. Tissue samples BLN, MLN, PGT, HL and LL were harvested, immediately flash frozen in liquid nitrogen, and placed on dry ice before storage at − 80°C. BoHV-1 viral load was assessed using qPCR and was performed on BLN, MLN, PGT and lung tissue samples (data not shown). BoHV1 PCR was performed using the exopol EXOone oneMIX BoHV-1 kit (exopol, Spain), according to the manufacturer’s instructions.

### RNA extraction

Total RNA was extracted using the Qiagen RNeasy Plus Universal Mini Kit (Qiagen LTD, Manchester, UK), according to the manufacturer’s instructions. The quantity of the extracted RNA was determined by measuring the absorbance at 260 nm with a Nanodrop spectrophotometer (Nano Drop technologies; Wilmington, DE, USA). The quality of the extracted RNA was examined with the Agilent 2100 Bioanalyzer (Agilent Technologies Ireland Ltd; Dublin, Ireland) using the RNA 6000 Nano LabChip kit (Agilent Technologies Ireland Ltd; Dublin, Ireland). The mean ±  SD RNA Integrity Number for BLN, MLN, PGT, HL and LL samples were 9.07 ±  0.11, 9.32 ±  0.21, 9.4 ±  0.17, 8.83 ±  0.16 and 9.14 ±  0.30 respectively.

### RNA-Seq library preparation and sequencing

RNA samples were shipped frozen at − 80°C on dry ice to the University of Missouri’s Genomics Technology Core (Columbia Missouri, USA). Library preparation was performed using the TruSeq stranded mRNA kit (Illumina, San Diego, California, USA) and libraries sequenced (150 bp (BLN, lung) or 100 bp (MLN, PGT) paired-end) on an Illumina NovaSeq 6000. All sequence data produced in this study have been deposited to the NCBI GEO repository and are available under the following accession codes: GSE153242 for BLN, GSE153429 for cranial lung lobe, GSE227344 for PGT and GSE229115 for MLN. Whole blood RNA-Seq data from O’Donoghue et al. (2023) are available at GEO with accession number GSE199108.

### Read processing

Sequence reads were received in FASTQ format and were assessed for quality using FastQC (version 0.11.8). Reads were trimmed at the 3’ end using cutadapt (version 1.18) [19] for the removal of short reads ( < 10 bases), ambiguous nucleotides and poly-G-artefacts. Trimmed reads were then re-assessed using FastQC to ensure that all reads passed the basic quality statistics. Sequence reads were aligned to the ARS-UCD1.2 bovine reference genome [20] and read counts generated using the STAR (Spliced Transcripts Alignment to a Reference) alignment tool (version 2.6.1b).

### Differential expression analysis

Raw read counts were input into R (version 4.3.2 (2023-10-31)) and differential expression analysis performed using the edgeR package (version 4.0.5) which uses an over-dispersed model to account for arising biological and technical variation [21]. Genes with less than one read count per million in the smallest number of samples per treatment were removed from the analysis. The trimmed-mean of M values method was used to normalise the data and the negative binomial dispersion parameters were estimated using the quantile-adjusted conditional maximum likelihood estimator. Differentially expressed (DE) genes between the treatment (control and challenged) groups were detected using exact tests, with genes considered DE if they had a Benjamini- Hochberg false discovery rate (FDR) of ≤  0.1 and a fold change of ≥  2. To analyse the DEGs common across tissues, DEGs from each tissue type were input into the Venn diagram tool on BioTools.fr https://www.biotools.fr/misc/venny. Genes common to all tissue types, which had the same direction of regulation (upregulated) between treatments were ranked using a methodology similar to that described by Laighneach *et al*. (2021) [22]. For each tissue, genes were ordered by statistical significance for DE and assigned a rank (with the most significant DEG ranked first). The rank for each DEG within each tissue type were then summed across tissues to produce a sum rank. A spearman correlation was performed using the cor() function in R (version 4.3.2 (2023-10-31)) to assess the relationship between viral load (qPCR data) and the common DEGs in each tissue type. Correlation (effect size and the strength of the correlation) was described using the following: 0.00–0.19 “very weak”; 0.20–0.39 “weak”; 0.40–0.59 “moderate”; 0.60–0.79 “strong”; 0.80–1.0 “very strong” [23,24].

### Pathway and gene ontology analysis

For the detection of perturbed biological pathways and gene ontologies, DEGs were input into the Database for Annotation, Visualization and Integrated Discovery (DAVID) [25,26]. The lists of DEGs identified within each tissue type were separately uploaded to DAVID and Kyoto Encyclopaedia of Genes and Genomes (KEGG) and gene ontology (GO) terms for biological processes (BP), cellular components (CC) and molecular functions (MF) were interrogated. Pathways and ontologies with a P ≤  0.05 and FDR ≤  0.05 were considered enriched for DEGs. This analysis was also performed for the DEGs found to be common to all tissue types.

### Gene co-expression analysis

For each tissue dataset, raw RNA-seq read counts were filtered for lowly expressed genes, by retaining only genes that had greater than one count per million in at least 12 samples (BoHV-1 challenged) or 6 samples (control). The filtered reads were normalised in edgeR using the trimmed mean of M – values normalisation method. Normalised counts were then Log2(x +  1) transformed in R. Filtered reads were input into R and weighted gene co-expression network analysis (WGCNA) was performed on the data for each tissue type individually using the WGCNA R package (version 1.72-5) which identifies modules (or networks) of co-expressed genes [27]. First, a similarity matrix was constructed to estimate the similarity between genes with similar expression profiles. The similarity matrix was next transformed into an adjacency matrix using a soft thresholding power corresponding to R^2^ ≥  0.8 [14]. The adjacency matrix, which encodes the strength of the connection between pairs of genes, was finally transformed into a topological overlap matrix (TOM) and the corresponding dissimilarity (distance) of genes was calculated to minimize the effects of noise and false associations [28]. Average linkage hierarchical clustering was applied to the TOM for each tissue dataset using the hclust function in R to produce a hierarchically clustered tree of genes for each tissue type. The dynamicTreeCut package (version 1.63-1) 10.32614/CRAN.package.dynamicTreeCut was then used to identify modules (groups of co-expressed genes). Module eigenegenes were calculated using the moduleEigengenes function in the WGCNA package, to quantify the co-expression similarity of the entire module. Modules with genes possessing highly similar expression profiles were merged based on their correlation and assigned a colour identifier by the software [27].

Pearson’s correlation was used to analyse the relationship between identified modules of co-expressed genes and selected clinical traits (treatment (BoHV-1-challenged), clinical score and rectal temperature). A positive Pearson correlation indicates that a module is associated with BoHV-1 infection and a negative Pearson correlation indicates that a module is not associated with BoHV-1 infection. Modules with statistically significant (P ≤  0.05) correlations were selected for further analysis as biologically interesting modules associated with BoHV-1 infection.

### Identification of hub genes within modules of interest

The module membership (MM) can take a positive or negative value, indicating that genes are positively or negatively related to the module eigengene, respectively. Genes which have a MM value closer to an absolute value of 1 are highly connected to the other genes within a module (Langfelder and Horvath, 2008). We considered genes with MM >  0.9 to be hub genes [29]. Hub genes identified in the significant modules for each tissue data set were then cross-analysed against the identified DEGs to reveal the genes identified in both analyses.

### Pathway analysis and functional enrichment of modules of interest

Pathway analysis was conducted on genes present in modules significantly associated with the examined traits (BoHV-1-challenged, clinical score and rectal temperature). To perform these analyses the list of genes from each significant module were input into DAVID. The databases queried through DAVID were the KEGG, GO_BP, GO_CC and GO_MF. DAVID employs Fisher’s exact test to determine the enrichment of pathways and ontology terms. Significant pathways and terms were considered those with a P-value <  0.05 and FDR <  0.05.

### Protein-protein interaction (PPI) analysis

Genes that exist in close proximity to each other in a genome can have functional associations [30]. DEGs common to all tissue types were input into the search tool for recurring instances of neighbouring genes (STRING) (version 12.0) [30,31], for the examination of functional associations between their encoded proteins.

## Results

### Clinical scores, haematology variables and lung pathology

Clinical scores and lung pathology measures have been described previously [12]. Briefly, there was a significant treatment ×  day (d) interaction for both clinical scores (P <  0.05) and rectal temperature (P <  0.01) between control and challenged calves, whereby clinical scores and rectal temperatures were greater for BoHV-1 challenged compared to control calves on d 4, 5, and 6 post-challenge. BoHV-1 challenged calves had higher clinical scores (P <  0.001) and rectal temperatures (P <  0.0001) on d 3, 4, 5, and 6 relative to d -1. There were no differences in lung scores in the overall lung or the right cranial lung lobe lung between BoHV-1 challenged and control calves (P >  0.05).

### Differential gene expression analysis

The mean sequence reads ±  SD obtained for the tissues were; 40,815,975 ±  4,282,489 (BLN), 44,308,241 ±  4,896,483 (MLN), 81,200,000 ±  8,920,000 (PGT), 41,181,735 ±  4, 308, 861 (HL) and 40,502,064 ±  5,048,646 (LL) of which 94%, 86%, 86%, 94% and 94% of reads were uniquely aligned to the ARD UCD 1.2 bovine reference genome respectively.

**Fig 1:**
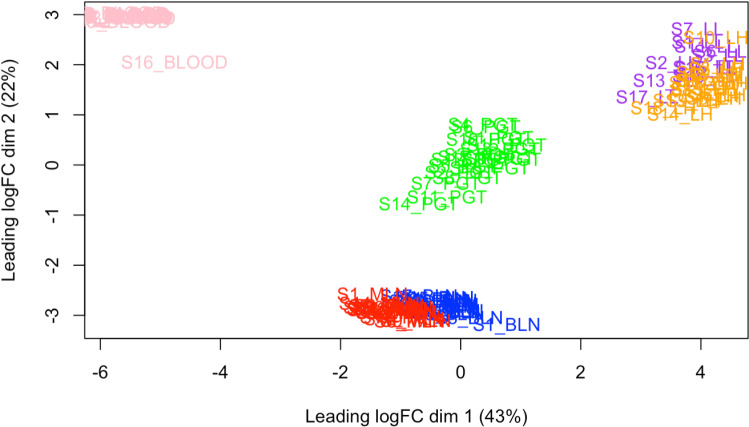
A multi-dimensional scaling plot based on all samples from each tissue type. Samples separated into four clusters; whole blood (pink), PGT (green), MLN and BLN (red and blue, respectively) and LH and LL (orange and purple, respectively).

There were 337, 81, 1833, 334, and 67 DEGs identified (P <  0.05, FDR <  0.1, FC >  2) between control and challenged calves in the BLN, MLN, PGT, HL and LL samples respectively (S1 Table). Multi-dimensional scaling based on global gene expression, revealed a clear separation between the control and challenged groups in the BLN, PGT and HL (Supplementary Figures 1–5 S2 File). Multi-dimensional scaling of all samples based on global gene expression revealed four distinct clusters (Fig 1). The gene expression profiles of the whole blood and PGT separated into two distinct clusters. The MLN and BLN samples formed a cluster, as did samples from the LH and LL.

**Fig 2:**
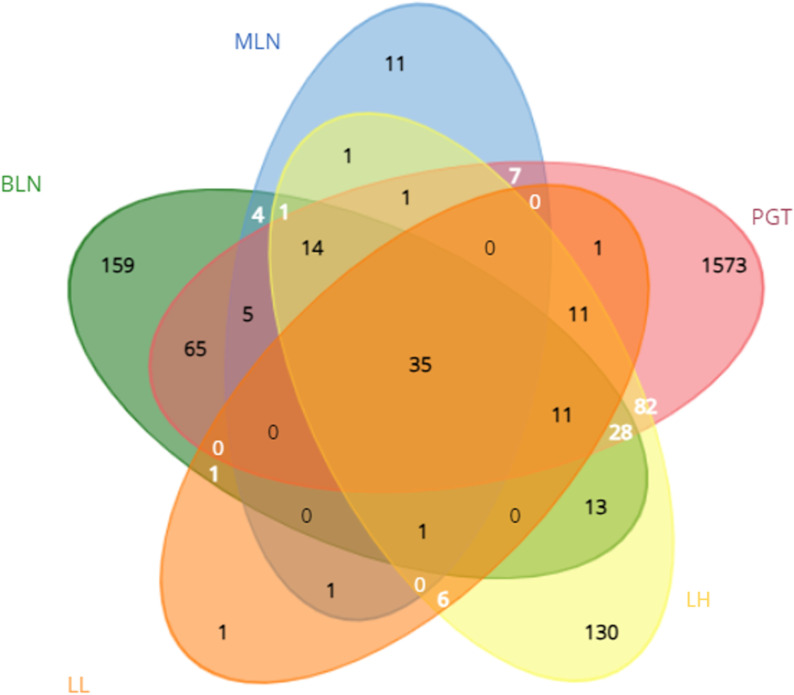
An overview of the number of genes commonly DE between tissues types in response to BoHV-1 challenge. The corresponding colour and label for each node are; green, bronchial lymph node (BLN), blue, mediastinal lymph node (MLN), pink, pharyngeal tonsil (PGT), yellow, healthy lung (LH) and orange, lesioned lung (LL).

A Venn diagram was used to display the DEGs in common across tissue types (**Fig 2**). There were 35 DEGs common to all tissue types, and all were upregulated in response to BoHV-1 (S2 Table). Strikingly, thirty-three of these genes were also DE and upregulated in the whole blood data from O’Donoghue *et al*. (2023). **Table 1** shows the sum ranks of p-values for these DEGs across all tissues with the lowest sum rank representing the most significant gene.

**Table 1:** The sum-ranked DEGs common across all tissue types (BLN, MLN, PGT, LH, LL and whole blood), in response to a BoHV-1 challenge in dairy calves. DEGs are ordered based on rank with the lowest ranked gene (most significant) listed first. Ensemble ID’s, gene symbol and an associated function is shown for each gene.

ENSEMBLE ID	Gene Symbol	Associated Function	Sum Rank
ENSBTAG00000012406	*ZBP1*	An innate immune sensor involved in the activation of the interferon response [32]. Plays a role in the induction of inflammatory cell death (pyroptosis, apoptosis and necroptosis) [33].	49
ENSBTAG00000019018	*IFITM3*	Interferon inducible membrane protein that restricts viral infection [34].	54
ENSBTAG00000020166	*ZNFX1*	Encoded protein is a zinc finger protein involved in the innate and antiviral immune response [35].	55
ENSBTAG00000003366	*RIGI*	Involved in the recognition of viral RNA [36].	60
ENSBTAG00000053806	ENSBTAG00000053806	Upregulated in the bovine endometrium in response to interferon-tau [37].	60
ENSBTAG00000017367	*IFIT5*	Involved in innate and anti-viral immune responses [38].	65
ENSBTAG00000014628	*OAS2*	Interferon-induced gene with a vital role in the antiviral activity of interferons [39].	73
ENSBTAG00000030932	*IFI44L*	Involved in the negative modulation of innate immune response to viral infection. Excessive immune responses can be harmful to hosts, therefore negative feedback is needed [40].	77
ENSBTAG00000039861	*OAS1Y*	Interferon-induced gene with a vital role in the antiviral activity of interferons [39].	79
ENSBTAG00000037527	*OAS1X*	Interferon-induced gene with a vital role in the antiviral activity of interferons [39].	85
ENSBTAG00000046580	*DHX58*	Involved in the negative regulation of RIG-1 signalling and type 1 interferon production [41].	93
ENSBTAG00000020536	*HERC6*	Encodes proteins induced by type I interferons, and is upregulated in cattle with BRD [42].	97
ENSBTAG00000052306	*ENSBTAG00000052306*	Unknown	98
ENSBTAG00000019018	*IFITM3*	Interferon inducible membrane protein that restricts viral infection [34].	98
ENSBTAG00000008471	*MX2*	Involved in the innate immune response to viral infection [43].	99
ENSBTAG00000010057	*GZMB*	Encodes a member of the granzyme subfamily of proteins involved in the defence response to viral infection [44].	100
ENSBTAG00000025398	ENSBTAG00000025398	Amongst the most DE hub genes associated with intramuscular fat in Nellore cattle [45].	103
ENSBTAG00000011343	*XAF1*	Targets IRF7 to inhibit type 1 interferon production during viral infection [46].	107
ENSBTAG00000008021	ENSBTAG00000008021	DE in the endometrium of pregnant dairy cows 15 days after insemination [47].	110
ENSBTAG00000007554	*IFI6*	Involved in the negative modulation of innate immune response [48].	110
ENSBTAG00000045588	ENSBTAG00000045588	Function unknown; Upregulated in bovine endometrium explants in response to interferon-tau and conceptuses [49].	111
ENSBTAG00000003152	*IFI27*	Role in viral infections. Counterbalances the innate immune response to RNA viral infection [50].	111
ENSBTAG00000021565	*PRSS2*	Downregulated in Holstein-Friesian cell lines infected with *Theileria annulata* [51].	121
ENSBTAG00000030913	*MX1*	Interferon-induced protein with antiviral activities [52].	122
ENSBTAG00000008703	*EIF2AK2*	Encodes a serine/threonine kinase involved in type 1 interferon production and the regulation of transcription factors including NF- kB and IRF-1 [53,54].	123
ENSBTAG00000014707	*ISG15*	Interferon-induced ubiquitin like modifier with a role in the host response to viral infection [55].	123
ENSBTAG00000007881	*IFIT1*	Interferon stimulated gene involved in the innate immune response [56].	124
ENSBTAG00000016061	*RSAD2*	Interferon stimulated gene with role in the innate and antiviral immune responses [57].	133
ENSBTAG00000019979	*CMPK2*	Involved in inflammatory and viral respiratory disease [58]; Role in antitumor and antibacterial immunity [59].	136
ENSBTAG00000014529	GBP4	Upregulated in non-classical and intermediate monocytes in *Bos taurus* cattle [60]. GBP4 is a negative regulator of virus-triggered IFN-1 production through the targeting and inhibition of IRF7 [61].	138
ENSBTAG00000012335	*UBA7*	Involved in activating ISG15 for antiviral functioning [62].	139
ENSBTAG00000032265	*RTP4*	Involved in antiviral immune response [63].	151
ENSBTAG00000053807	ENSBTAG00000053807	DE in the endometrium of pregnant dairy cows 15 days after insemination [47].	162

Significant relationships were found between the expression of these genes and the BoHV-1 viral load in the PGT and MLN, with 19 and 1 gene(s) moderately significantly correlated respectively (S1 File).

### PPI analysis

**Fig 3:**
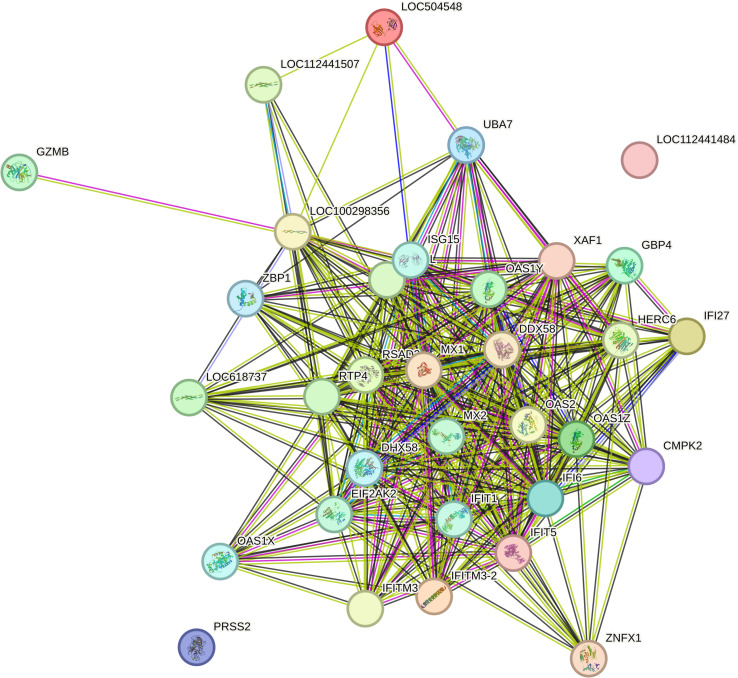
A PPI network for the 33 DEGs common to all tissue type. Nodes represent each protein where coloured nodes represent the first shell of interactors and white nodes represent second shell of interactors. Within some nodes the known or predicted 3D structure is given and in blank nodes the 3D structure of the protein is unknown. Edges represent the protein-protein associations and are colour coded; light blue =  known interactions from curated databases, pink =  known interactions determined experimentally, green =  predicted interactions gene neighbourhood, red =  predicted interaction gene fusion, dark blue =  predicted interaction gene co-occurrence. The light green, black and lilac edges represent other interactions such as text mining, co-expression and protein homology respectively.

An examination of the functional connections among the proteins encoded by the 33 genes common to all tissue types in STRING (version 12) revealed that this gene group had significantly more interactions than expected if the genes had been sampled at random (P < 1.0e-16). This suggests that these proteins are biologically connected as a group. A PPI network for these genes is presented in **Fig 3** (S3 Table). **Table 2** presents the STRING functional analysis of the PPI network.

**Table 2:** Functional analysis conducted in STRING for the 33 DEGs common across all tissue types.

Biological Process (Gene Ontology)				
Term/cluster no:	Description	Count in network	Strength	FDR
GO:0060700	Regulation of ribonuclease activity	4 of 9	2.47	1.56E-06
GO:0070106	interleukin-27-mediated signalling pathway	2 of 5	2.42	0.0135
GO:032020	ISG15-protein conjugation	2 of 6	2.34	0.0168
GO:0045071	Negative regulation of viral genome replication	14 of 45	2.31	2.03E-25
GO:0034340	Response to type I interferon	7 of 26	2.25	2.39E-11
Molecular Function (Gene Ontology)				
GO:0001730	2-5-oligoadenylate synthetase activity	4 of 5	2.72	1.5E-06
GO:0008191	Metalloendopeptidase inhibitor activity	3 of 17	2.07	0.0027
GO:0003725	Double-stranded RNA binding	7 of 73	1.8	1.03E-07
GO:003723	RNA binding	11 of 1191	0.79	0.00081
Cellular Component (Gene Ontology)				
GO:0005737	Cytoplasm	28 of 10284	0.26	0.0139
Local network cluster (STRING)				
CL:18979	Dynamin, GTPase and Host cell	5 of 5	2.82	5.70E-10
CL:18976	ISG15 antiviral mechanism, and TLD	7 of 10	2.67	7.54E-14
CL:18969	Negative regulation of viral genome replication and cellular response to interferon-alpha	14 of 21	2.65	1.51E-29
KEGG Pathways				
bta05160	Hepatitis C	10 of 150	1.65	8.7E-12
bta5164	Influenza A	10 of 160	1.62	8.7E-12
bta05162	Measles	8 of 136	1.59	3.85E-09
bta04622	RIG-I-like receptor signalling pathway	3 of 65	1.49	0.0069
bta05169	Epstein-Barr virus infection	7 of 197	1.37	1.59E-06
Reactome Pathways				
BTA-1169408	ISG15 antiviral mechanism	6 of 26	2.18	1.04E-08
BTA-936440	Negative regulators of DDX58/IFIH1 signalling	3 of 17	2.07	0.0014
BTA-168256	Immune system	8 of 802	0.82	0.0211
WikiPathways				
WP1017	Type II interferon signalling (IFNG)	4 0f 30	1.95	4.61E-05
Tissue Expression (TISSUES)				
BTO:0000801	Macrophage	4 of 102	1.41	0.0211
BTO:0000089	Blood	6 of 427	0.97	0.0225
BTO:0000570	Hematopoietic system	14 of 1106	0.92	5.64E-07
BTO:0005810	Immune system	8 of 802	0.82	0.0211
Annotated Keywords (UniProt)				
KW-0051	Antiviral defence	6 of 51	1.89	1.25E-07
KW-0399	Innate immunity	5 of 113	1.47	0.00022

The term number provides a numerical reference to each term or pathway.

For each term, a description as well as the count in network is provided. The latter is a count of the number of genes from our data out of the total number of genes associated with that term.

The strength figure is the Log10 (observed/expected), and describes how large the enrichment is, based on the number of proteins in our network annotated with the term and the number of proteins expected to be annotated in a random network of the same size.

The FDR details the p-value for each term corrected for multiple testing using the Benjamini-Hochberg procedure.

### Pathway and gene ontology analysis

**Fig 4:**
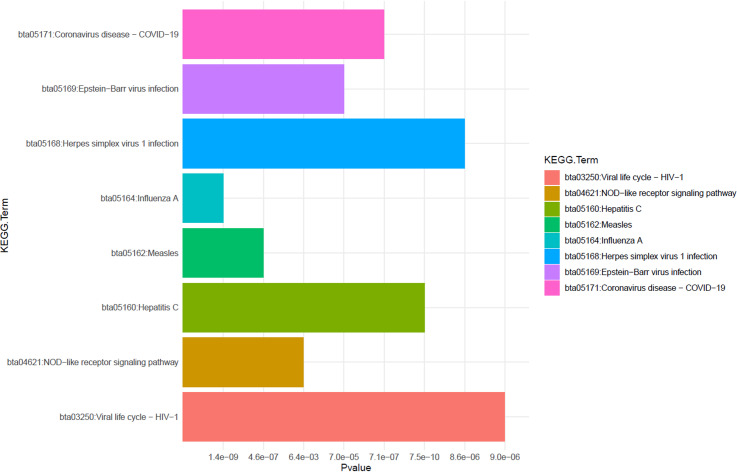
A bar plot displaying the KEGG pathways enriched for the DEGs common to all tissue types. The significance (P-value) for each term is given on the X-axis and the term name is given on Y-axis.

Analysis of the DEGs from each tissue type identified pathways and ontology terms associated with the innate and antiviral immune responses across tissue types (S4–S8 Tables**).** Analysis of the 33 DEGs common to all tissue types revealed enriched pathways related viral diseases such as Influenza A, Hepatitis C and Herpes simplex virus 1 infection. Other identified pathways were related to pathogen recognition receptor signalling such as NOD-like receptor signalling (S9 Table). **Fig 4** displays the KEGG pathways enriched for the 33 DEGs common to all tissue types.

### Tissue specific weighted gene co-expression analysis

After filtering, there were a total of 14341, 14205, 14760, 14908, 14690 and 14743 genes remaining in the BLN, MLN, PGT, LL, LH and blood data sets respectively, for downstream analysis. A soft-thresholding power as determined by the scale-free topology of each network (R^2^ >  0.8) was used to construct the adjacency matrix. The blood, LL and PGT datasets failed to reach a scale-free topology fit index above 0.8 and so a power of 9 was used for each of these analyses, as advised by the package information https://github.com/edo98811/WGCNA_official_documentation/blob/main/faq.html. A soft-thresholding power of 20 (BLN), 10 (MLN) and 9 (PGT, LH, LL and blood) were used for each analysis. Modules with a correlation >  0.5 and P ≤  0.05 were considered significantly associated with each analysed trait, in this case, treatment (BoHV-1 challenge), clinical score and rectal temperature.

WGCNA identified modules significantly associated with BoHV-1 infection in each tissue dataset, and in some cases modules significantly associated with clinical score and rectal temperature were also identified. A heatmap detailing the modules significant for each tissue type is provided in S6 Fig. A complete list of the genes within each significant module for BLN, MLN, PGT, LH, LL and whole blood are provided in S10–S15 Tables, respectively.

### Pathway analysis of genes within modules

Analysis of the genes from each significant module for each tissue type through DAVID revealed enriched pathways and gene ontology terms (P <  0.05, FDR <  0.05). There were no significant KEGG pathways identified for the genes in significant modules in the BLN or MLN data. A list of the KEGG pathways enriched for significant modules in the PGT, LH, LL and whole blood data are provided in S16–S19 Tables, respectively.

**Fig 5:**
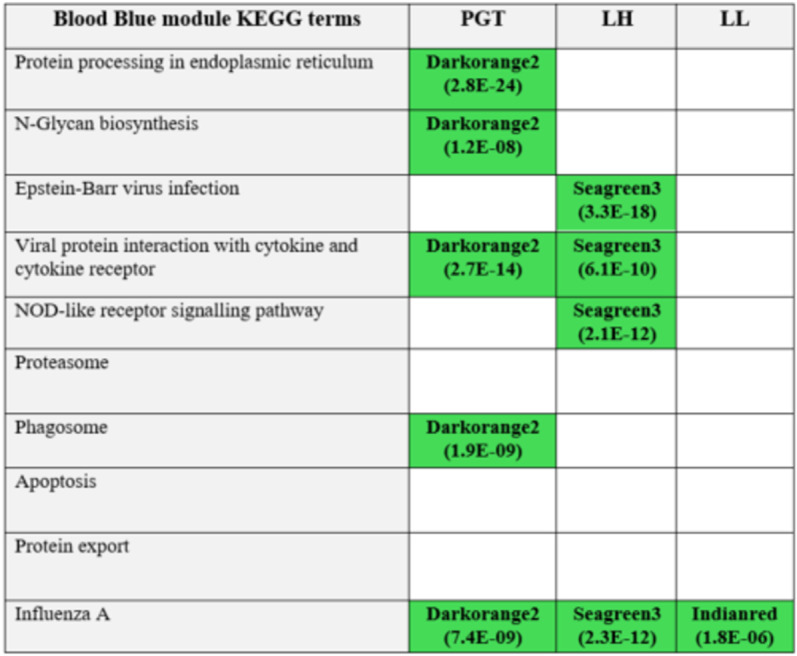
A matrix detailing the overlap of KEGG pathways enriched for genes in the blue module of the blood and the other tissues. Each KEGG term is listed on the left with the tissue codes given across the top of each column. The green squares represent the terms from the blue module that overlap in that tissue type. The module within each tissue is given in each green square as well as their respective P-value (in brackets).

The blue module identified for the blood, was significantly and positively associated with BoHV-1 infection. Many of the KEGG pathways enriched for the genes in this module were also enriched for genes in significant modules across the other tissue types. **Fig 5** shows the KEGG pathways from this module that are also enriched in other tissue modules.

### Hub gene identification and overlap with DEGs

**Fig 6:**
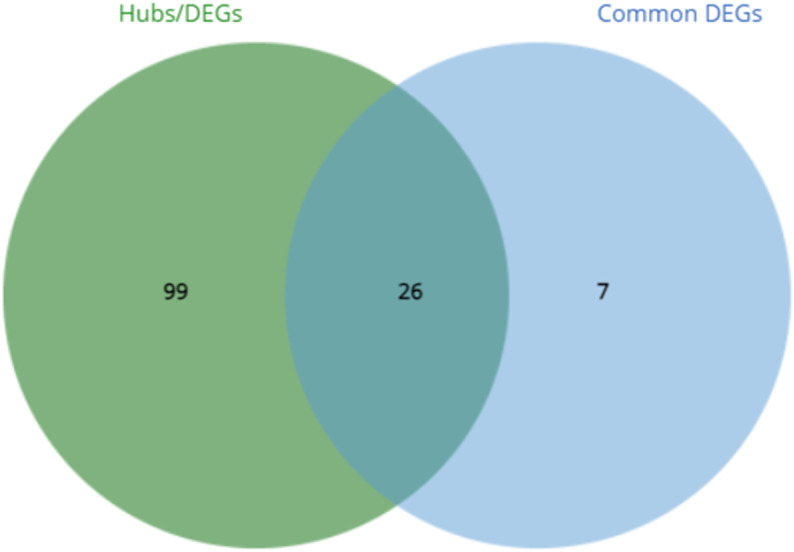
A Venn diagram showing the overlap of genes from the blood blue module identified as hubs and DEGs with those common across all tissue types.

Across all the tissue types, 3472 hub genes (MM >  0.9) were identified. A list of the hub genes identified for each tissue type is provided in S20 Table. The blue module identified in the blood analysis had many overlaps in enriched pathways with significant modules across other tissue types. Five hundred and seventeen hub genes (MM >  0.9) were identified in this module, with 125 also identified as DEGs by O’Donoghue *et al*. (2023). Of these, 26 were amongst the 33 genes common to all tissues (**Fig 6**), demonstrating that these genes are centrally important to the processes enriched in this module. A list of the hubs genes also found to be DE in each tissue type is provided in S21 Table.

## Discussion

In the present study, DE and WGCNA approaches were used to analyse the transcriptome profiles of bronchial, and mediastinal lymph node, pharyngeal tonsil, healthy and lesioned lung and whole blood samples collected from dairy calves experimentally infected with BoHV-1. Previous studies by our groups have examined the transcriptome response of various tissue types from animals following a single pathogen challenge with BRD causative agents (Tizioto et al., 2015; Behura et al., 2017; Johnston et al; 2019; Johnston et al., 2021; O’Donoghue et al; 2023). These studies used a functional genomics approach, RNA-Seq, to identify DEGs between different phenotypes (e.g., control versus infected animals). This methodology has been successful in uncovering genes and biological pathways involved in the host immune response to BRD, with our group identifying differentially regulated genes in response to experimental challenges with BRSV (Johnston et al., 2019; Johnston et al., 2021) and BoHV-1 (O’Donoghue et al, 2023). The use of co-expression networks for the examination of correlation patterns across tissues to find clusters of highly correlated genes and regulatory genes within these networks is crucial [18,27], given that genes and gene products operate in association with many other genes, connected in larger, more complex networks. For example, Behura et al. (2017) utilised a network approach to identify key genes and networks enriched in various lung and lymphoid tissue in beef calves following experimental challenge with a range of viral and bacterial BRD causative agents. Other studies have utilised network-based analyses to investigate the regulatory networks in whole blood that may be involved in naturally contracted BRD infection [13–15]. In studies of naturally infected animals, clinical signs are often used as a means of BRD diagnosis, making it difficult to identify the causative agent and thus, the initial cause of infection.

We found 33 DEGs in common among all of the analysed tissues in response to BoHV-1 infection. The upregulation of all these genes in all tissues in response to infection suggests that they play a key role in mounting the across-tissue immune response to BoHV-1. Furthermore, the fact that these genes are also upregulated in circulating whole blood suggests that they may have potential as ‘ante-mortem’ biomarkers of BRD, allowing earlier diagnosis of BoHV-1 infection. Many of these genes (*DHX58*, *EIF2AK2*, *GZMB*, *IFI44L*, *IFI6*, *IFITM3*, *ISG15*, *MX1*, *MX2*, *OASIY*, *OAS2* and *RSAD2*) were also identified as hub-hub genes in the whole blood of cattle with BRD and were suggested to be critical in the immune response to BRD infections [14]. Further, Johnston et al. (2019) found several of these genes (*EIF2AK2*, *IFIT1*, *IFIT5*, *ISG15*, *MX1*, *OAS2* and *RSAD2*) to be DE in the bronchial lymph node of dairy calves following an experimental challenge with BRSV [64]. A study analysing the bronchial lymph node transcriptome profiles of beef cattle following single pathogen challenges with a range of BRD associated bacterial and viral pathogens also found some of these genes (*HERC6*, *ZBP1*, *IFIT1*, *ISG15* and *RSAD2*) to be DE in response to all challenge pathogens [11]. These findings also support the utility of these genes as potential biomarkers for the presence of BRD infection. Scott et al. (2022a) discussed the potential of rapid diagnostics for testing cattle for BRD upon arrival to the feedlot. Whole blood, as a tissue source, would be ideal for this application as collection is relatively non-invasive, economical and practical [65]. Moreover, the usage of antibiotics in herds might potentially be decreased with testing, which has applications in both the dairy and beef industries.

The blue module identified in the whole blood data was enriched for several KEGG pathways that were also activated in some of the other tissue types. The pathways for N-glycan biosynthesis and protein processing in the endoplasmic reticulum were enriched in this module and were also enriched for module genes identified for the PGT. Glycans are an essential part of cell-cell interactions and N-glycans have been described as fine-tuners of immunological responses and potential molecular targets for the manipulation of immune tolerance and activation in a wide range of diseases and pathological conditions [66]. N- glycans, although involved in the immune response, can also be involved in viral attachment and entry to cells [67].

Analysis of the clinical scores found differences between the BoHV-1 challenged and control calves, as previously described [12]. Disease severity was not included as a variable in the DE analysis, representing a possible study limitation. However, clinical score was included in the WGCNA with the antiquewhite2 and sienna3 module significantly correlated with this trait in the BLN and MLN tissues respectively. Despite being significantly correlated, no KEGG pathways or ontology terms were enriched for the genes or hub genes in either module.

The pathways for ‘Cytokine-cytokine receptor interaction’ and ‘Viral protein interaction with cytokine and cytokine receptor’ were the most significant for DEGs in the PGT and were also enriched for genes in modules identified in the blood, LH and PGT. Cytokines are non-structural proteins, which play a regulatory role in processes such as inflammation, immunity and haematopoiesis [68], and often act as protectors against dangerous stimuli such as invading pathogens [69]. As a strategy to evade the host immune response, large DNA viruses, such as herpesviruses, encode homologues of cytokines, chemokines and their receptors [70], targeting chemokine-signalling networks to disrupt immune surveillance and defence of vertebrates [71]. The molecular mimicry of cytokines and cytokine receptors is known to be an immune modulation strategy adopted by herpesviruses [71]. Glycoprotein G (gG) is encoded in alphaherpesviruses and homologues of gG were found in BoHV-1 [72]. Recombinant gG from BoHV-1 was found to bind to a broad range of chemokines with high affinity and inhibit their biological activity *in vitro* [73]. These findings illuminate the nature of host-virus interactions occurring during BoHV-1 infection.

The most highly upregulated gene across all tissues analysed in the current study was *PRSS2* or serine protease 2. This gene was identified as the second most upregulated gene in whole blood in response to BoHV-1 [12]. Serine proteases are involved in a diverse range of biological processes including cellular and humoral immunity [74]. Other serine protease genes have been found to be differentially regulated in BRD cases with *PRSS45* downregulated in feedlot cattle at arrival that went on to develop BRD [75] while *PRSS50* was upregulated in feedlot cattle with BRD [76]. In humans, *PRSS2* is involved in inflammatory diseases [77,78] as well as tumour growth [79]. A recent study found that *PRSS2* is a key gene involved the regulation of lipid metabolism in dairy cows [80]. We found *PRSS2* to be amongst the DEGs involved in the protein digestion and absorption pathway in addition to several collagen genes that were downregulated in the bronchia lymph node, which may suggest it plays a role in collagen degradation. Additionally, we found this gene was also involved in the Influenza A pathway in other tissues. The significant upregulation of this gene across all our datasets in response to BoHV-1 infection suggests that it plays a key role in response BoHV-1 infection in dairy calves, and further investigation of its mechanism of action is warranted. Validation of key DEGs through qRT-PCR would strengthen the claim that these genes are central to the immune response to BoHV-1 infection, and we acknowledge that the absence of this validation could be a study limitation. Several pathways related to various viral infections such as Influenza A and Herpes Simplex infection were also identified. The Herpes Simplex infection pathway was enriched for the DEGs common to all tissue types as well as for genes in several significant modules. BoHV-1 and HSV1 share several biological properties, with both initiating infection in mucosal surfaces [81] and both are able to establish latency in the host sensory neurons [82]. DEGs and the gene modules involved in this pathway provide insight into the genes associated with the response to BoHV-1 infection.

Upon infection, pathogens express molecules known as pathogen-associated molecular patterns, which are recognised by host sensors called pathogen recognition receptors [83]. Nucleotide-binding oligomerization domain-like receptors (NLRs) are one such family of these receptors involved in the host response to BRD [84] and play a critical role in pathogen recognition and immune signalling [85]. Retinoic acid-inducible gene I (RIGI-I) like receptors are RNA sensors crucial in innate antiviral immunity [86] and the detection of viral replication in the cytoplasm during early infection [87]. The NLR and RIG-I-like receptor signalling pathways were enriched for the DEGs common to all tissue types, suggesting that they play a role in the cross-tissue host response to infection, a finding similar to that of Behura et al. (2017). With their role in innate and adaptive immunity, and their influence on downstream signalling pathways, NLRs have been suggested as targets for therapeutic strategies for auto-inflammatory disorders [88]. In humans certain micro-RNAs have been shown to promote RIG-I-like signalling and enhance the antiviral immune response to certain viruses [89]. Genes involved in these pathways may serve as potential targets for BRD therapies.

Respiratory disease causing viruses often spread to the mediastinal lymph nodes from the lungs [90,91]. Of the identified DEGs, 11 were unique to this tissue type. Of these, the antimicrobial peptides *CATHL2* and *PGLYRP1* were also found to be upregulated in Holstein-Friesian cows with clinical mastitis [92]. The *AGRN* gene was also found to be uniquely upregulated in the MLN and a hypothesis surrounding its role in immune system regulation depicts that agrin expression in T cells may be upregulated during pathogenic infection and plays a role in cell-cell adhesion that is required for successful T cell activation [93]. The serine protease *PRSS45* was uniquely DE in the MLN and was found to be downregulated in the current study. Interestingly, this gene was found to be upregulated in the whole blood of cattle that had resisted BRD infection [75]. To our knowledge, this is the first study to examine the transcriptomic changes in the mediastinal lymph node in response to a BoHV-1 challenge in dairy calves.

We found the pharyngeal tonsil to possess the greatest number of DEGs in response to BoHV-1 infection, with 1833 genes DE. Further examination of these DEGs, revealed that 1573 ( ~ 86%) were unique to the PGT. The IL-17 signalling pathway was one of the most significant for the unique DEGs. Although we did not find an altered expression of *1L-17* in this tissue, we did detect the downregulation of both *IL17RE* and *IL17A*. In early stages of the host response to a virus, the host immune system induces the production of interferons and pro-inflammatory cytokines [94]. IL-17, which is primarily produced by γδ T cells and CD4 +  or Th17 cells [95], is a ‘master regulator’ of downstream cytokine and chemokine activities [96] and plays a key role in the maintenance of tissue integrity and the induction of protective immune responses to pathogens, particularly at epithelial barrier sites [97]. An increase in the expression of IL-17 was observed in the lungs of calves infected with the BRD–associated virus, BRSV [96]. Although a critical component of the host defence to viral infections, it has also been thought to play a role in the promotion of viral infection and related illness [98]. The ontology term for interleukin-27-mediated signalling pathway was also found to be enriched in the STRING analysis of the 33 DEGs common to all tissues. Interestingly, IL-17 has become a major drug target in a range of autoimmune and inflammatory diseases [99], and so could have potential as a target for BRD therapies.

The pathway for protein digestion and absorption was enriched in the BLN and many of the DEGs involved in this pathway (*ATP1A2*, *COL5A3*, *ELN*, *COL21A1*, *COL4A1*, *COL4A2*, *COL3A1*, *COL5A1* and *COL8A1*) were unique to this tissue type. All of these genes were found to be downregulated in response to BoHV-1 infection. A link exists between the extracellular matrix (ECM) and innate and adaptive immunity and the degradation and deposition of collagen is related to immune cell activity [100]. Collagen degradation in the extracellular matrix is known to result from tissue damage during infection and can enhance inflammation [100]. Interestingly, degraded collagen has also shown to be a chemoattractant for immune cells [101]. The unique differential expression of these genes in the BLN suggest this tissue to be involved in a specific aspect of the overall immune response to BoHV-1.

The right cranial lung lobes are a primary site for lesion development during BoHV-1infection. Despite appearing healthy, we were able to identify over 300 DEGs in the LH, of which 130 were unique to this tissue. Genes in the phospholipase A2 group (*PLAG27* and *PLA2G2D5*) were found to be unique to this tissue and were upregulated in response to BoHV-1. Phospholipases are enzymes responsible for the cleavage of ester bonds within phospholipids [102] and have been shown to play a role in host defence against bacteria, parasites and viruses [103]. *IRF9* was also uniquely upregulated in the healthy lung and is involved in virus-mediated activation of interferon [104]. In humans, it was found that mutations in *IRF9* resulted in increased susceptibility to viral infection and that IRF9 deficient cells were unable to induce multiple interferon-stimulated genes (ISGs) [105]. This could suggest that *IRF9* plays a role in the regulation of ISGs in the lung during BoHV-1 infection.

## Conclusion

Our use of a controlled experimental challenge allowed for an improved understanding of the specific immune responses to BoHV-1. Building on the work of others in this field, this study has successfully identified DEGs and important gene networks associated with BoHV-1 infection. *PRSS2* was found to be highly upregulated in all analysed tissues and therefore warrants further investigation into its role in BoHV-1 infection. A better understanding of the biological mechanisms underlying the host response to BoHV-1 was made possible by the application of a systems biology approach as opposed to a DE analysis alone. The discovery of DEGs that are present in all tissue types, including whole blood, suggests prospective future targets for BRD therapies and diagnostics.

## Supporting information

S1 Table A list of the DEGs identified for each tissue type. (XLSX)

S2 Table A list of the DEGS in each section of the VENN diagram. (XLSX)

S3 Table A PPI network of the 33 DEGS common to all tissue types. (XLSX)

S4 Table The DAVID results for the BLN tissue. (XLSX)

S5 Table The DAVID results for the MLN tissue. (XLSX)

S6 Table The DAVID results for the PGT tissue. (XLSX)

S7 Table The DAVID results for the HL tissue. (XLSX)

S8 Table The DAVID results for the LL tissue. (XLSX)

S9 Table The DAVID results for the 33 DEGs common to all tissues. (XLSX)

S10 Table A list of the genes in the significant modules in the BLN. (XLSX)

S11 Table A list of the genes in the significant modules in the MLN. (XLSX)

S12 Table A list of the genes in the significant modules in the PGT. (XLSX)

S13 Table A list of the genes in the significant modules in the LH. (XLSX)

S14 Table A list of the genes in the significant modules in the LL. (XLSX)

S15 Table A list of the genes in the significant modules in the blood. (XLSX)

S16 Table Enriched KEGG pathways for genes in the significant modules in the PGT. (XLSX)

S17 Table Enriched KEGG pathways for genes in the significant modules in the LH. (XLSX)

S18 Table Enriched KEGG pathways for genes in the significant modules in the LL. (XLSX)

S19 Table The DAVID results for genes in the significant modules in the blood. (XLSX)

S20 Table The hub genes identified for each tissue type. (XLSX)

S21 Table A list of the hubs genes also found to be DE in each tissue type. (XLSX)

S1 File Spearman correlation results. (XLSX)

S2 File Figures 1-5. MDS plots from the DE analysis performed for each tissue type. (DOCX)

S6 Figure Module trait association heatmaps for the modules detected in each tissue type (A =  BLN, B =  MLN, C =  PGT, D =  HL, E =  LL and F =  Blood). The module-trait relationships were colour coded based on correlation between the module and trait with red =  strong positive correlation and green =  strong negative correlation. The correlation coefficient and p- value (in parentheses) are given for each association. The traits are listed along the X-axis (treatment, clinical score and rectal temperature) and each module colour is given on the Y-axis. (TIF)
