# Supplementary material for: Examination of the lung and lymphoid tissue mRNA transcriptome response in dairy calves following experimental challenge with bovine alphaherpesvirus one (BoHV-1)
Source: PLoS One. 2025 May 2;20(5):e0319575. doi: 10.1371/journal.pone.0319575 (PMC12047826; doi:10.1371/journal.pone.0319575)
Supplement: S2 File — (DOCX) [file pone.0319575.s023.docx]

**Supplemental Information for:**

**Examination of the lung and lymphoid tissue mRNA transcriptome response in dairy calves following experimentally challenge with bovine alphaherpesvirus one (BoHV-1)**

**Stephanie O’Donoghue^1,4^, Bernadette Earley^1^, Dayle O. Johnston^1^, Matthew S. McCabe^1^, Louise S. Cosby^2^, Ken Lemon^2^, Michael McMenamy^2^, Jeremy F. Taylor^3^, JaeWoo Kim^3^, Derek W. Morris^4^ and Sinéad M. Waters^4^***

^1^Animal and Bioscience Research Department, Animal and Grassland Research and Innovation Centre, Teagasc, Grange, Co. Meath, Ireland

^2^Veterinary Sciences Division, Agri-Food and Biosciences Institute, Stormont, Belfast, Northern Ireland

^3^Division of Animal Sciences, University of Missouri, Columbia, MO, USA

^4^Discipline of Biochemistry, University of Galway, Galway


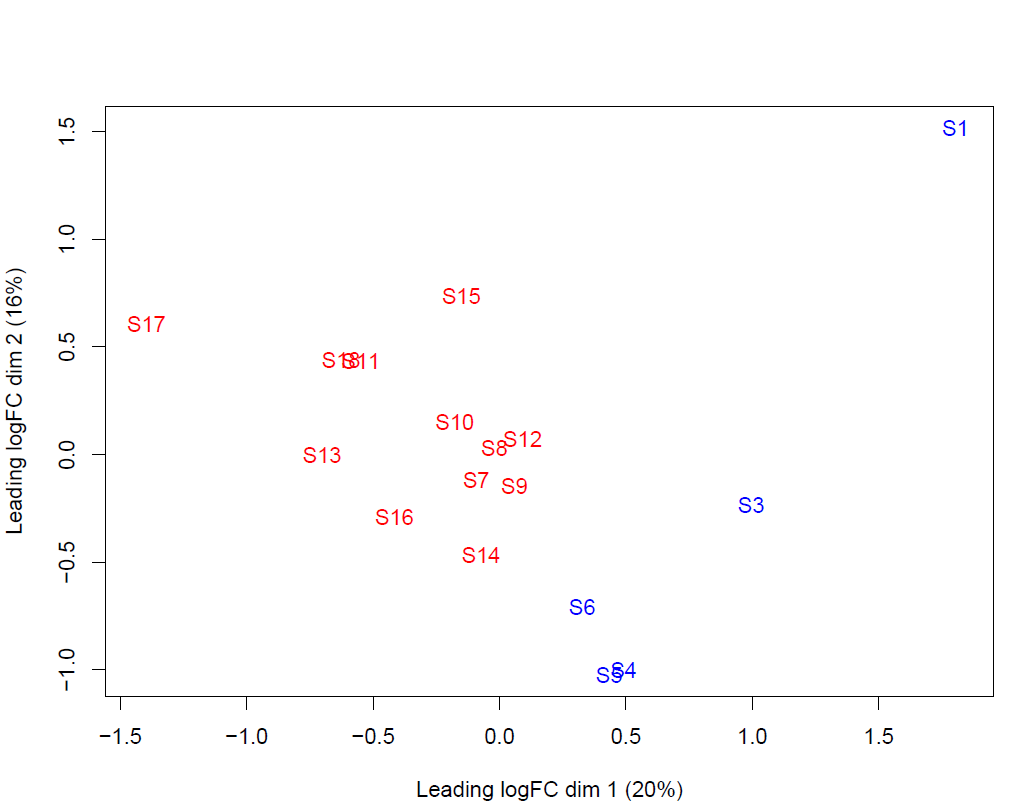


**Supplementary Figure 1:** An MDS plot generated in the edgeR R package displaying the separation based on global gene expression of **bronchial lymph node** tissue from control and BoHV-1 challenged calves. Samples from the Challenge calves are coloured red and Control samples are coloured blue. The numbers (1-18) refer to the calf ID. Sample (S); S1-S6 (Control) S7-S18 (Challenge).


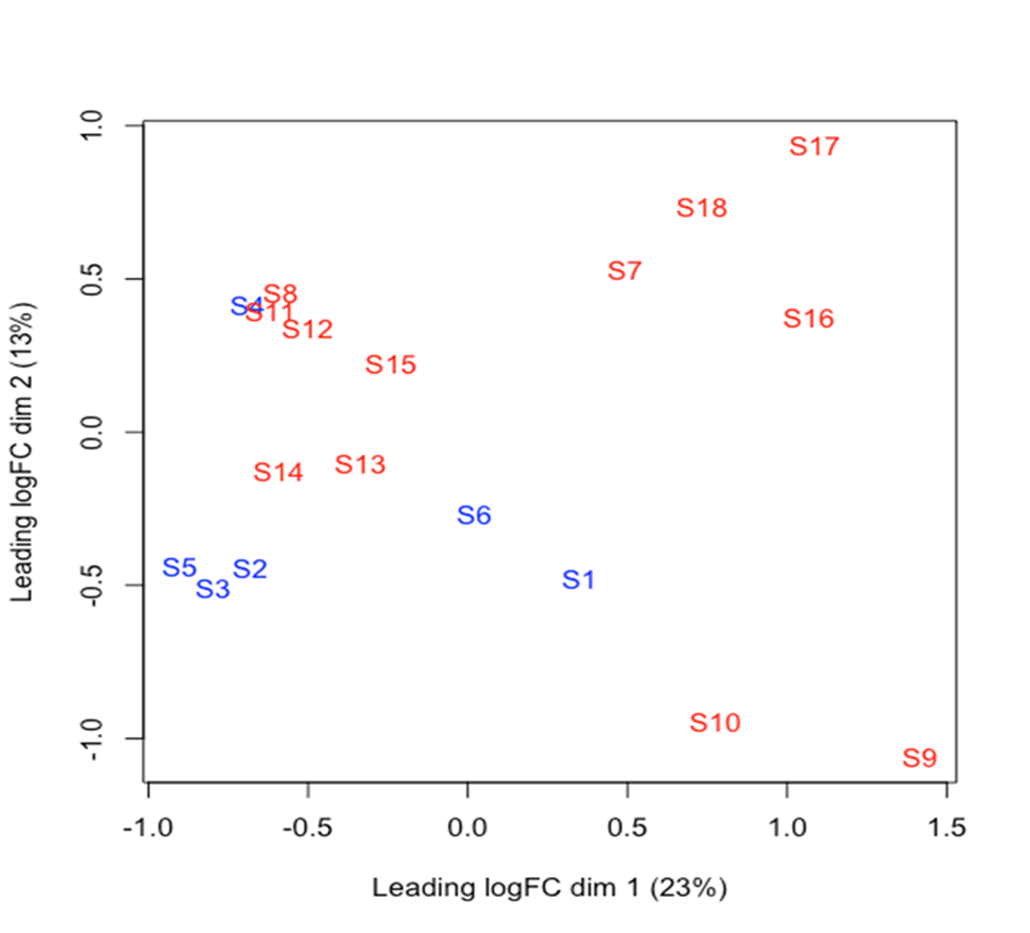


**Supplementary Figure 2:** An MDS plot generated in the edgeR R package displaying the separation based on global gene expression of **mediastinal lymph node** tissue from control and BoHV-1 challenged calves. Samples from the challenge calves are coloured red and control samples are coloured blue. The numbers (1-18) refer to the calf ID. Sample (S); S1-S6 (Control) S7-S18 (Challenge).


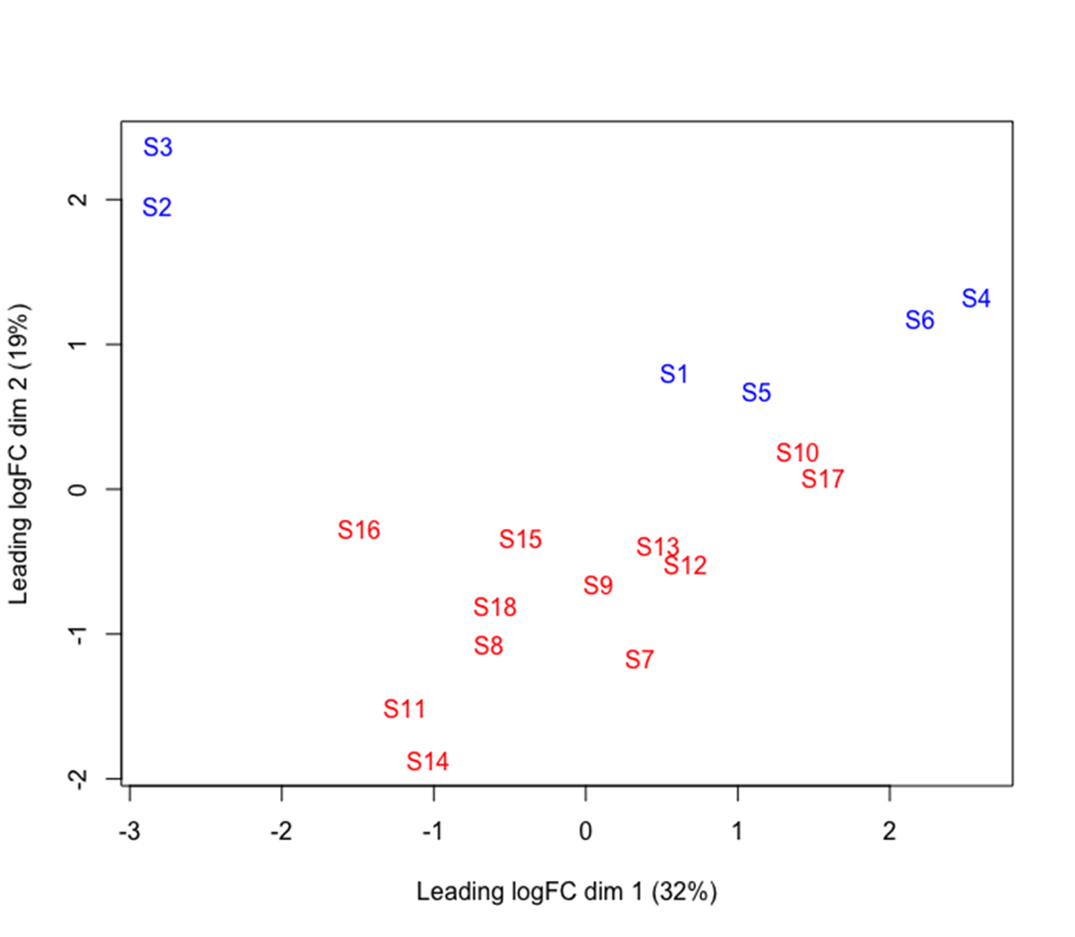


**Supplementary Figure 3:** An MDS plot generated in the edgeR R package displaying the separation based on global gene expression of **pharyngeal tonsil** tissue from control and BoHV-1 challenged calves. Samples from the challenge calves are coloured red and control samples are coloured blue. The numbers (1-18) refer to the calf ID. Sample (S); S1-S6 (Control) S7-S18 (Challenge).


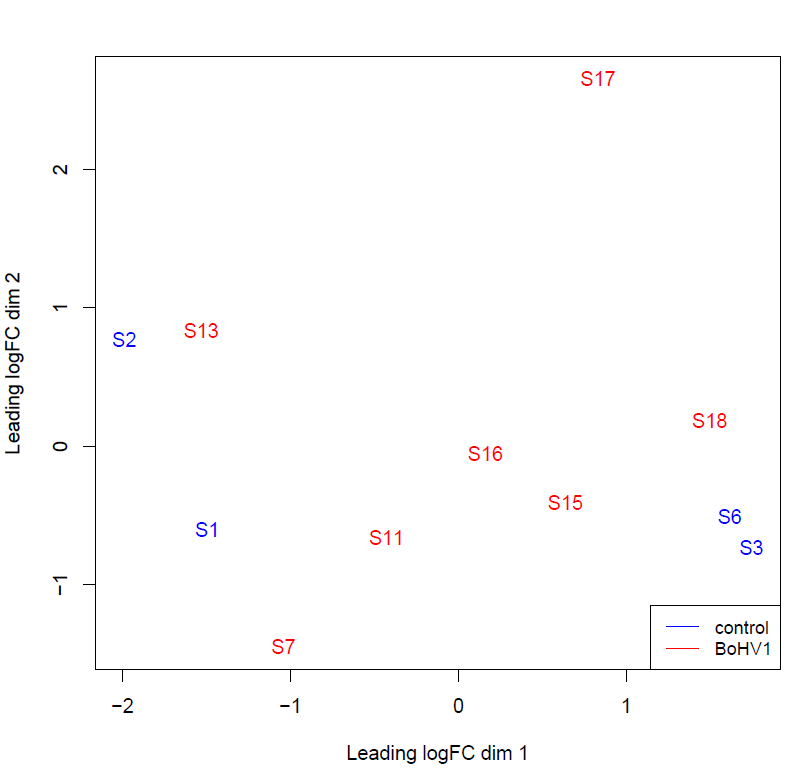


**Supplementary Figure 4:** An MDS plot generated in the edgeR R package displaying the separation based on global gene expression of **lesioned right cranial lung lobe** tissue from control and BoHV-1 challenged calves. Samples from the challenge calves are coloured red and control samples are coloured blue. The numbers (1-18) refer to the calf ID. Sample (S); S1-S6 (Control) S7-S18 (Challenge).


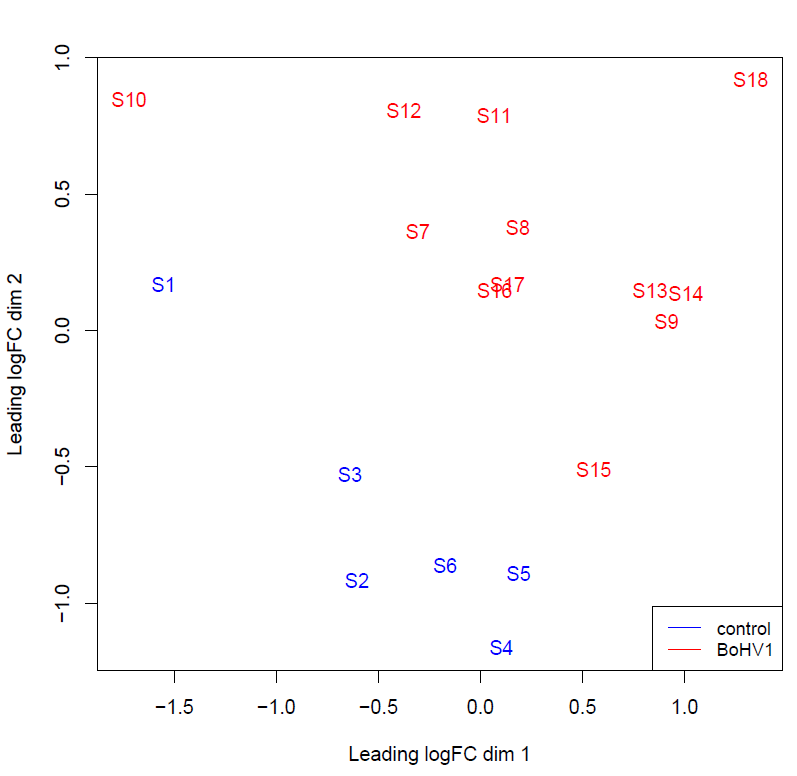


**Supplementary Figure 5:** An MDS plot generated in the edgeR R package displaying the separation based on global gene expression of **healthy right cranial lung lobe** tissue from control and BoHV-1 challenged calves. Samples from the challenge calves are coloured red and control samples are coloured blue. The numbers (1-18) refer to the calf ID. Sample (S); S1-S6 (Control) S7-S18 (Challenge).
