# Supplementary figures and images for: Examination of the lung and lymphoid tissue mRNA transcriptome response in dairy calves following experimental challenge with bovine alphaherpesvirus one (BoHV-1)
Source: PLoS One. 2025 May 2;20(5):e0319575. doi: 10.1371/journal.pone.0319575 (PMC12047826; doi:10.1371/journal.pone.0319575)

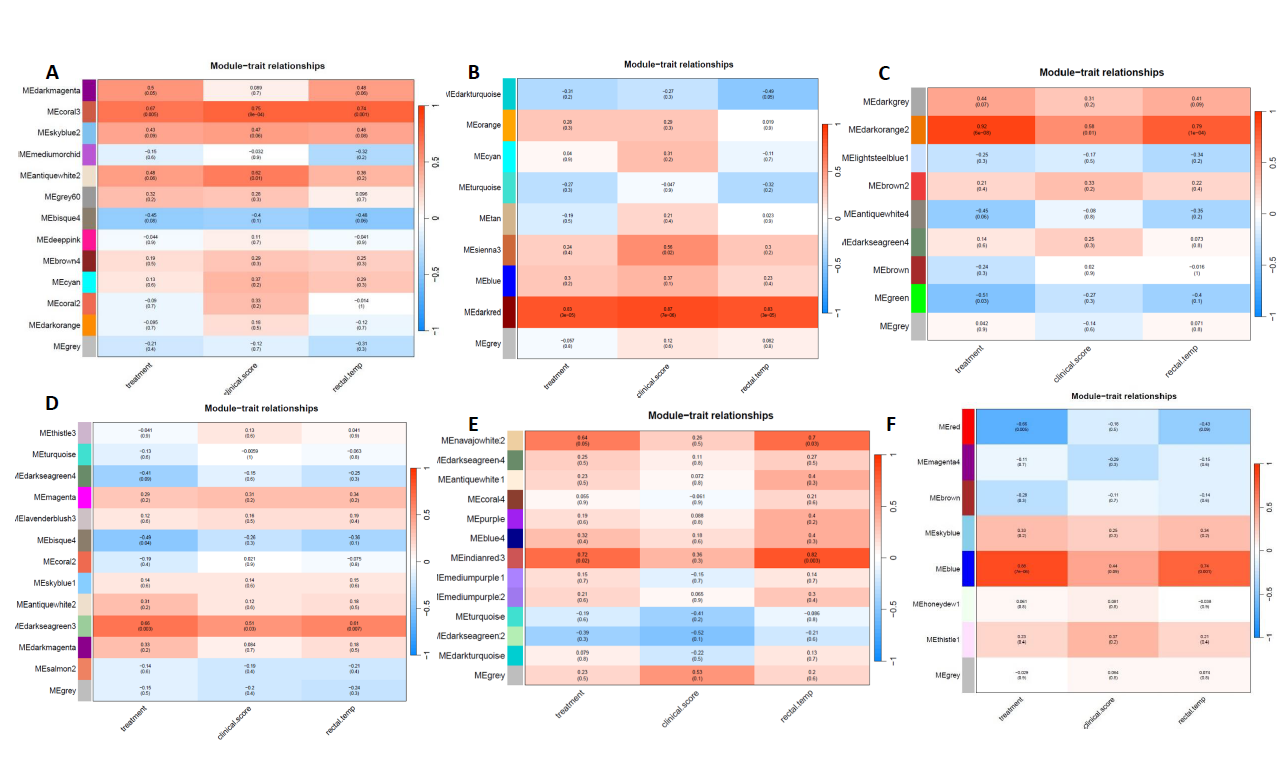

Supplement: S6 Figure — (TIF) [file pone.0319575.s024.tif]
